# Professor Paine’s Defence of the Medical Profession of the United States

**Published:** 1846-05-01

**Authors:** 


*Professor Paine's Defence of the Medical Profession of the United States.*—This is the title of a valedictory address to the graduating class at the medical commencement of the University of New York. The subjects of the address are, in fact, the proposed national convention, and the projector of the movement, Dr. Davis, of Binghampton. Dr. D., it appears, as chairman of a committee appointed by the State Society on the subject of the convention, and in some remarks published in the New York Journal of Medicine, has animadverted on the existing state of the profession as regards education and acquirement, in rather strong terms. Prof. Paine chooses to consider this in the light of a calumnious assault on the character of the profession, and himself called upon valiantly to take up the gauntlet in its defence. This explains the title of the address.

On the whole, it is a very curious production. Considering the occasion, the selection of subjects denotes a singular taste; and the performance itself, for a professorial address, is unique. They who are somewhat acquainted with the previous publications of the writer, however, may not be so much surprised at its peculiarities, as if it were the first specimen of the literary taste and abilities of the writer, with which the public has been favored.

It seems that the chairman of the committee appointed by our State Medical Society to correspond on the subject of the convention, in his official letter to the University of New York, committed two or three errors of orthography. At least, so it is made to appear by a printed copy of the letter, with certain errors italicised. Whether they were from carelessness, or whether the Professor himself committed the blunders in decyphering the manuscript, or whether, having a multitude of letters to write, Dr. D. employed an amanuensis, or, lastly, whether it be really true that the projector of the convention be but an indifferent speller, we will not take it upon ourselves to decide. We leave it for Dr. Davis to substantiate his claims to the character of a good orthographer. But for a venerable professor in a metropolitan school, to descend to such puerilities in a public discourse, on such an occasion, is more than undignified—it is pitiful and contemptible in the extreme.

Prof. P. absolves his colleagues from all knowledge of the topics which he selected, and all responsibility in his peculiar views. They have reason to be obliged to him for this acknowledgement. It does not appear that the address was solicited for publication, but it seems to have been printed solely upon the author's own instance. If we have been correctly informed as to its reception by the auditory before which it was delivered, its publication may be considered to be an appeal from the judgment of those who listened to its delivery to the decision of its readers.

We have alluded but to a single point of the address. It is abundantly open to criticism in other, and, indeed, in all points; but we do not think that our readers will be edified by an extended analysis.

We have some personal interest in the *argumentum ad hominem* which we have noticed. We have ourselves had the hardihood to give utterance to an opinion that the existing condition of our profession is far from being all that is to be desired. The last deduction, however, which we could have suspected would have been considered logical or appropriate by any person is, that by the expression of such an opinion one is to be considered as assuming aught on the score of his own attainments. We cannot think it necessary to argue in deprecation of this method of reasoning, as we cannot but believe that it belongs to the peculiar dialectics of the Professor of the institutes in the university of the city of New-York.

